# Inclusion of carotenoids in dietary habits as an alternative to prevent age-related macular degeneration

**DOI:** 10.3389/fnut.2022.1063517

**Published:** 2023-01-09

**Authors:** Iván Antonio García-Montalvo, Diana Matías-Pérez, Emilio Hernández-Bautista, Eduardo Pérez-Campos

**Affiliations:** Division of Graduate Studies and Research, National Institute of Technology of Mexico/Technological Institute of Oaxaca, Oaxaca, Mexico

**Keywords:** carotenoids, age-related macular degeneration (AMD), dietary habits, stress oxidative, lifestyle

## Introduction

Age-related macular degeneration (AMD) is considered the leading cause of irreversible blindness in people between 55 and 65 years of age, and its prevalence is expected to increase in developed and developing countries, in proportion to the existing geriatric population (1–4). Its evolution is due to a complex interaction between demographic, racial, age, genetic, and environmental factors, i.e., AMD is multifactorial. Age is the factor with the highest degree of association with this pathology, followed by smoking as a trigger for the release of free radicals that directly affect the retina. Frequent alcohol consumption leads to a deficient absorption of nutrients, mainly water-soluble vitamins. Other factors involved are poor or no antioxidant intake, obesity, hypertension, prolonged exposure to the sun, ethnicity, and family history (5). These visual disturbances have a great impact on the lifestyle of patients, compromising their daily activities (driving or reading difficulties) (5).

## Age-related macular degeneration and carotenoids

AMD is considered a bilateral ocular pathology that damages the central part of the retina, called the macula lutea. It is responsible for photopic vision, which allows visualization of fine details and image resolution. The fovea is in the center of the macula lutea, where the photoreceptor cells are concentrated. Drusens are deposits of cellular debris and are the most common feature of this disease along with the development of choroidal neovascularization (6, 7).

Abnormalities present in photoreceptors, Brunch's membrane, pigment epithelium, and choroid result in neovascularization and geographic atrophy (8). According to the fundoscopic abnormalities observed, they can be classified into two general phenotypes: wet (exudative), and dry (atrophic) (8, 9). The dry phenotype (geographic atrophy) is characterized by progressive obstruction of the drusen, which causes thinning of the pigment epithelium and prevents proper oxygen transport to photoreceptors (10). The wet phenotype can be divided into choroidal neovascularization and polypoidal choroidal vasculopathy, which may involve blood or serum leakage. There are cases in which there is an evolution from dry to wet phenotype (11–13). As mentioned above, dietary habits have been identified as risk factors involved in the origin or development of diseases but can be susceptible to modification. It is important to include foods with antioxidant properties in the regular diet to reduce the constant flow of oxidizing molecules that lead to the development of various maculopathies, including AMD (14). Some risk or pathophysiological aspects to consider in this pathology are stimulation of the complement system, loss of homeostasis between pro-inflammatory and anti-inflammatory factors, activation of microglia and genetic susceptibility (15, 16). It should be considered that the retina has an oxygen rich environment, in addition to prolonged exposure to light, leading to the continuous production of free radicals (17). To reduce this damage to the retinal epithelium, there are enzymatic mechanisms that maintain homeostasis (18). Due to aging, the retina has a weakened antioxidant system, making it susceptible to oxidation, manifesting itself as hard exudates in the periphery, thickening of Brunch's membrane, and thinning of the choriocapillaris (15). Nutritional intervention through the consumption of antioxidants, polyunsaturated fatty acids of plant and animal origin, carotenoids (lutein and zeaxanthin), and minerals has been shown to be effective in preventing or delaying the progression of AMD due to its involvement in decreasing inflammatory and oxidative events (19, 20). Among the main carotenoids that have demonstrated functionality in reducing AMD (see Table 1) are lutein, zeaxanthin, beta-carotene, crocin, and crocetin. Lutein/zeaxanthin supplementation improves visual performance, including glare tolerance, contrast sensitivity, and recovery from photostress (21–25). Crocin and crocetin are used as antiischemic, hypolipidemic, antihypertensive, anxiolytic, antidiabetic, antidepressant, anticancer, and cardioprotective agents (26–35). Antioxidants are used due to their ability to decrease/eliminate anti-inflammatory activity through the release of pro-inflammatory cytokines by glial cells, as well as regulation in inflammatory pathways (28).

**Table 1:** Clinical trials on the treatment of AMD and carotenoid consumption.

**Study design**	**Sample size**	**Dose**	**Results**	**Authors**
Multicenter epidemiologic follow-up study of the AREDS2 clinical trial.	3,882 subjects	AREDS2 supplementation with lutein/zeaxanthin (10 mg/2 mg), vitamins C (500 mg) and E (400 international units), and zinc (80 mg) plus copper (2 mg).	Lutein/zeaxanthin was a suitable substitute for beta-carotene in AREDS2 supplements. Compared with beta-carotene use, lutein/zeaxanthin had a potentially beneficial association with late AMD progression.	Chew et al. (22)
Randomized, unmasked, parallel-arm study.	27 subjects	Consumption of 28 g of goji berries or consumption of commercial supplement of lutein (6 mg) and zeaxanthin (4 mg).	90 days of goji berry consumption is associated with an increase in MPOD in healthy middle-aged adults. This may indicate that lutein, zeaxanthin, and other bioactive compounds in goji berries may be involved in increasing MPOD.	Li et al. (24)
Multicenter, randomized, observer-blinded study.	109 subjects	AREDS supplement (Theavit^®^), vitamin C (120 mg); vitamin E (20 mg); beta-carotene (800 mg); zinc (15 mg); manganese (2 mg) and selenium (50 μg). AREDS supplement (Retilut^®^), except beta-carotene, plus copper (2 mg), DHA (400 mg), lutein (10 mg), zeaxanthin (2 6 mg), resveratrol (30 mg) and hydroxytyrosol (3 mg).	There was reduction of inflammatory cytokines and improvement of fatty acid profile as well as serum lutein and zeaxanthin concentration. In patients with unilateral wet AMD, the addition of lutein, zeaxanthin, resveratrol, hydroxytyrosol and DHA at AREDS EU recommended doses in the short term had no differential effect on visual acuity compared to a standard AREDS EU formulation, but in addition to improving the fatty acid profile and increasing serum carotenoid levels, may provide a beneficial effect in improving the proinflammatory and proangiogenic profile of AMD patients.	García-Layana et al. (25)
Randomized, double-blind, placebo-controlled study.	115 subjects	Supplement with lutein (10 mg) and zeaxanthin (2 mg) or equivalent placebo.	Daily supplementation with lutein and zeaxanthin significantly increases their serum levels, increases MPOD, improves chromatic contrast and recovery from photo-stress.	Hammond et al. (21)
Randomized, double-blind, placebo-controlled study.	60 subjects	Saffron supplement (15 mg saffron extract) or placebo.	Daily supplementation with 30 mg of saffron for 6 months significantly improves retinal function in patients with AMD in the medium term.	Lashay et al. (35)

## Discussion

The lutein and zeaxanthin present in the retina is more abundant than in any other type of human tissue, however, their distribution is not uniform. In the fovea, the concentration of lutein is lower than that of zeaxanthin (1:2 ratio) (36–38) and decrease as they approach the ocular periphery. The generated production of reactive oxygen species (ROS) through extramitochondrial oxidative phosphorylation in the outer segment of rods (39, 40) leads to the release of compounds such as cytochrome c, which results in the mechanism of photoreceptor apoptosis (activation of caspase 9). The main function of lutein in the retina is to scavenge free radicals, due to its chemical structure in which there are two hydroxyl groups that are mainly responsible for trapping ROS (41–43). Xanthophylls and carotenes are the most effective oxygen regulators of the carotenoid family due to the double bonds present in their structures that facilitate the reduction of their energy state (36). Oxidative stress is caused by an imbalance between the two reactive species (ROS and RNS). These reactive species are a fundamental part of innate immunity that protects cells from infections and, unfortunately, contribute to the pathogenesis of degenerative diseases such as AMD. Under physiological conditions, the eye is sensitive to this type of stress due to the rich content of polyunsaturated lipids present in cell membranes and exposure to pro-oxidant agents.

We believe that the consumption of carotenoids provides benefits in the progressive improvement of AMD due to the antioxidant power they possess. Lutein and zeaxanthin can absorb specific wavelengths of light (490–495 nm) which helps in protecting the eyes. In addition to the fact that carotenoids may provide protection against certain types of cancer by limiting abnormal cell growth and improving communication between functional spaces, carotenoids may prevent heart disease by blocking the formation and oxidation of low-density lipoproteins. Ocular carotenoids (mesozeaxanthin, zeaxanthin and lutein isomers) are concentrated in the macula and fovea. Increasing their daily intake would provide benefits to ocular health, as carotenoids have several functions in the retina, such as protection against photochemical injury, ROS neutralizing capacity and protection against UV-induced peroxidation and reduction of lipofuscin formation. Despite the dosage trials that have been carried out and the results obtained, there are still gaps to be filled, considering the results of the trials consulted we can indicate that the intake of supplements with lutein/zeaxanthin content (10 mg/2 mg per day) is recommended over those supplements with beta-carotene, since the excessive use of beta-carotene is associated with the risk of lung cancer. Such is the case of the establishment of reference values by gender, age, and ethnicity worldwide, as well as the reclassification of these carotenoids, which should be considered as conditionally essential nutrients. This is the case of resveratrol present in grape seed oil and alpha-lipoic acid, which have been shown to be effective in reducing the loss of retinal ganglion cells and inhibiting the destructive processes that accompany them, with the aim of delaying degenerative eye diseases. We hope that the advancement and development of future research in this area can be considered as a contribution to global public health as we face a continuously increasing geriatric population.

## Author contributions

IG-M, DM-P, EH-B, and EP-C participated in the design, structuring, and revision of the manuscript. All authors contributed to the article and approved the submitted version.
